# Supplementary material for: Covalent transfer of chemical gradients onto a graphenic surface with 2D and 3D control
Source: Nat Commun. 2022 Nov 16;13:7006. doi: 10.1038/s41467-022-34684-w (PMC9668971; doi:10.1038/s41467-022-34684-w)
Supplement: Supplementary file 1 — Supplementary information [file 41467_2022_34684_MOESM1_ESM.pdf]

Supplementary Information

Covalent transfer of chemical gradients onto a graphenic surface with 2D and 3D control

Yuanzhi Xia[1]†, Semih Sevim[2]†, João Pedro Vale[3,4]†, Johannes Seibel[1], David Rodríguez-San-Miguel[5], Donghoon Kim[2], Salvador Pané[2], Tiago Sotto Mayor[3,4]*, Steven De Feyter[1]*, Josep Puigmartí-Luis[5,6]*

[1]*Department of Chemistry, Division of Molecular Imaging and Photonics, KU Leuven, Celestijnenlaan 200F, B-3001 Leuven, Belgium.*

[2]*Multi-Scale Robotics Lab, Institute of Robotics and Intelligent Systems, ETH Zurich, Tannenstrasse 3, CH-8092 Zurich, Switzerland.*

[3]*Transport Phenomena Research Centre (CEFT), Engineering Faculty of Porto University, Rua Dr. Roberto Frias, 4200-465 Porto, Portugal*

[4]*Associate Laboratory in Chemical Engineering (ALiCE), Engineering Faculty of Porto University, Rua Dr. Roberto Frias, 4200-465 Porto, Portugal*

[5]*Departament de Ciència dels Materials i Química Física, Institut de Química Teòrica i Computacional, University of Barcelona (UB), 08028 Barcelona, Spain*

[6]*Institució Catalana de Recerca i Estudis Avançats (ICREA), Pg. Lluís Companys 23, 08010 Barcelona, Spain*

† These authors contributed equally: Yuanzhi Xia, Semih Sevim, João Pedro Vale

E-mail: *tiago.sottomayor@fe.up.pt, steven.defeyter@kuleuven.be, josep.puigmarti@ub.edu*

Table of Contents

Supplementary Figures 3

Supplementary Tables 21

Supplementary Notes 22

Supplementary Note 1: Calculation of diffusion coefficient with DOSY for numerical simulations 22

Supplementary Note 2: Reaction mechanism 22

Supplementary Note 3: Calculation of reaction kinetics with UV-Vis spectroscopy for numerical simulations 22

Supplementary Note 4: Analysis of flow in the main microfluidic chamber for flow rates of 10 to 600 μL/min 23

Supplementary Note 5: Screening of reaction rate constants that fit the experimental data of layer thicknesses 24

Supplementary References 26

Supplementary Figures

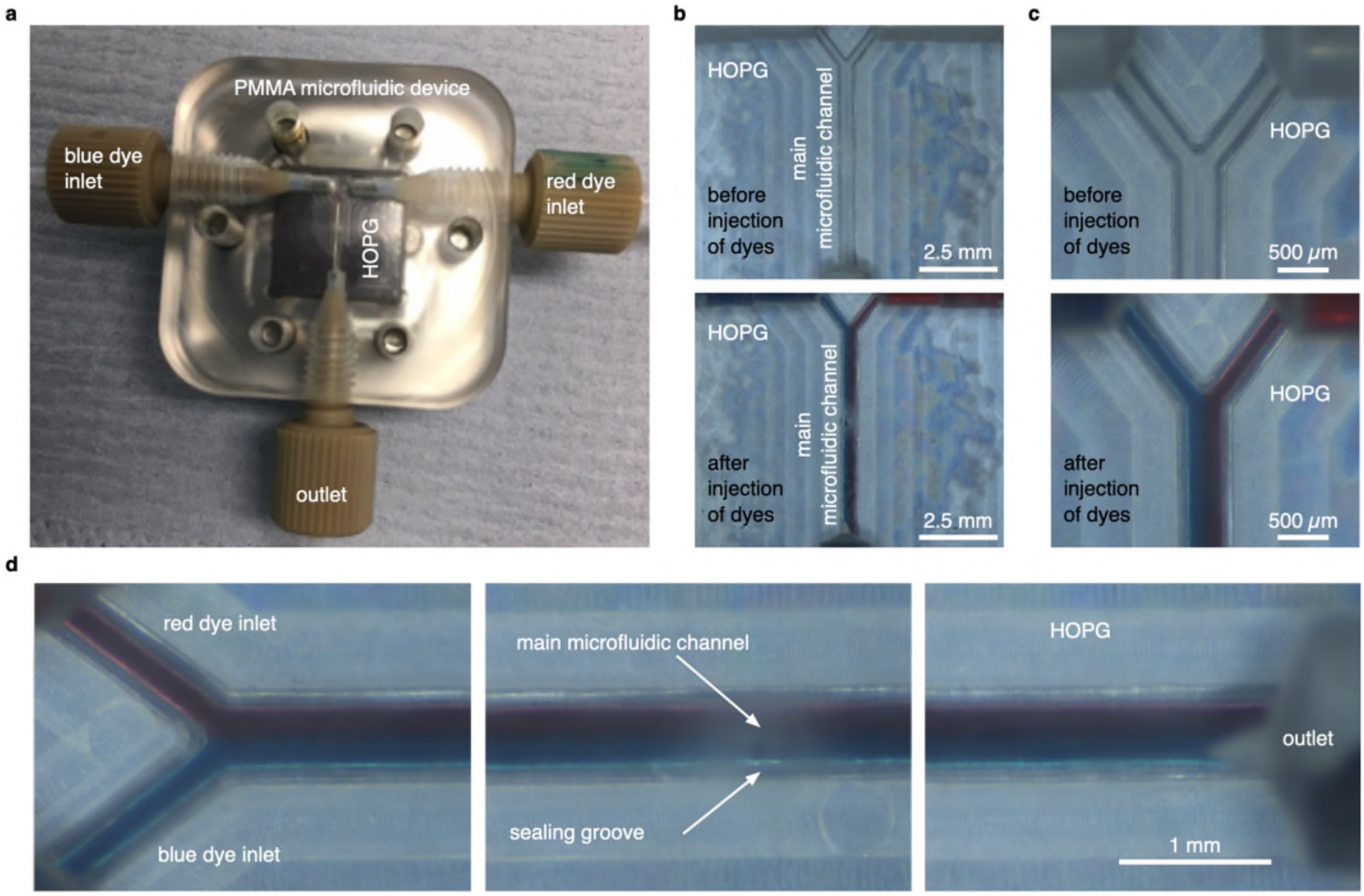

Supplementary Fig. 1. Laminar flow over the HOPG substrate. a, Photograph of the assembled microfluidic device. **b,** Microscope images showing the entire microfluidic network before (on top) and after (on bottom) injection of food dyes. In **c,** zoom-in micrographs of the inlet part. **d,** Magnified images of the Y-shaped microfluidic channel showing the red and blue food dyes coflowing over the HOPG substrate without any leakage. Scale bars: 2.5 mm in **b**, 500 μm in **c** and 1mm in **d**.

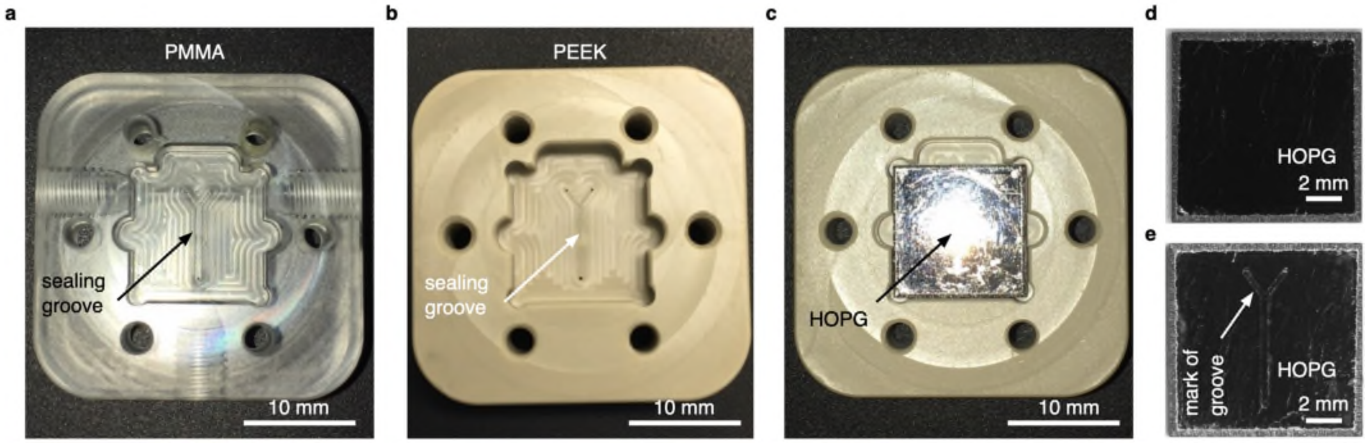

Supplementary Fig. 2. Machined top and bottom layers. Micrographs of the top layer made of **a,** poly(methyl methacrylate) (PMMA) and **b,** polyether ether ketone (PEEK). In both cases the sealing groove around the microfluidic network is apparent. **c,** PEEK top layer integrated with the HOPG substrate. **d-e,** 12 mm x 12 mm HOPG substrate **d,** before mechanical clamping and **e,** after the experiment with the mark of the sealing groove created on surface due to the mechanical clamping. Note that the marks generated on the HOPG substrate by the sealing groove allows for mapping out the positions of different zones and areas within these zones, while performing the characterization of the grafted surfaces. Scale bars: 10 mm in **a-c**, and 2 mm in **d-e**.

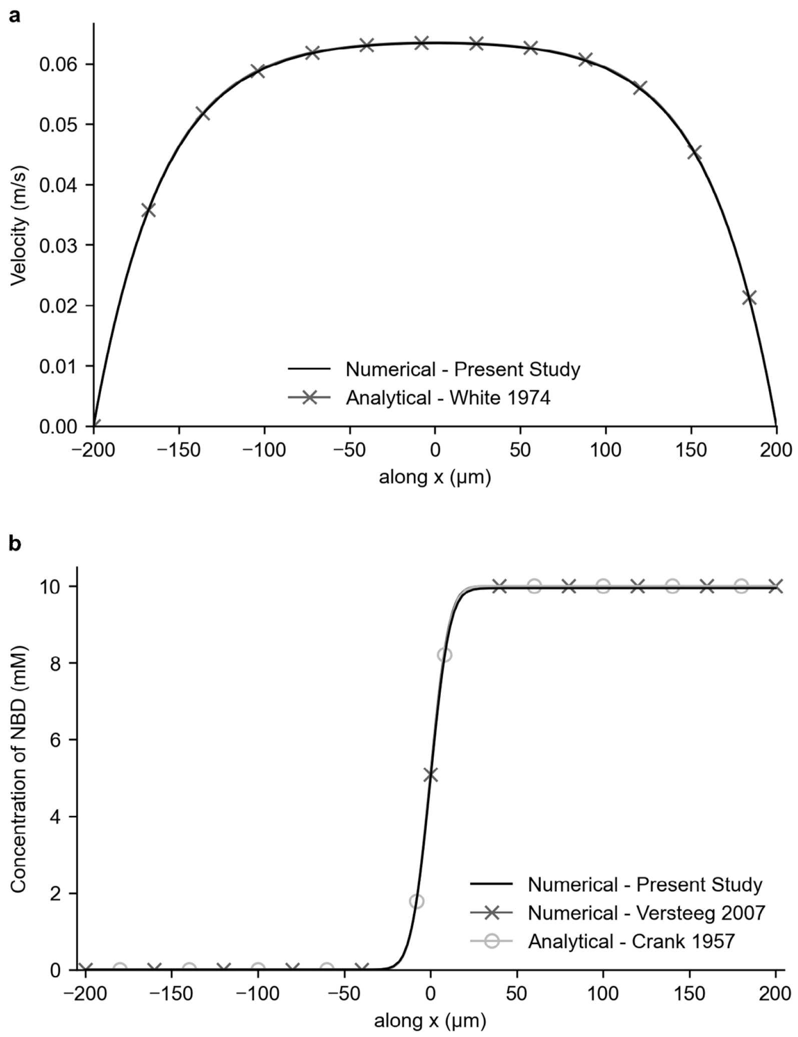

Supplementary Fig. 3. Validation of the flow and mass transport in the microfluidic device simulations. a, Velocity profile along x in zone 2 obtained by numerical simulation in the present study (full black line) and from analytical solution[1] (crosses). **b,** Concentration profile of the NBD diffusion along x in zone 2 obtained by numerical simulation in the present study (full black line), from a 1D numerical solution of the diffusion equation[2] (crosses) and from an analytical solution[3] (circles).

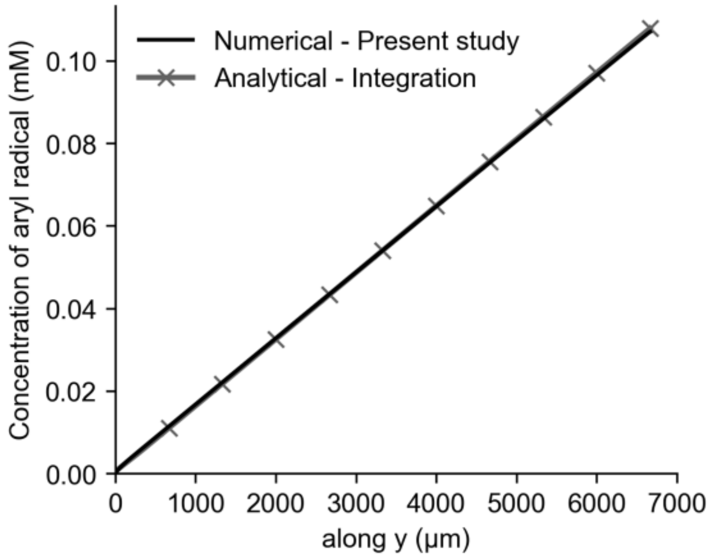

Supplementary Fig. 4. Predictions of aryl radical concentration continuously produced along the channel, obtained by the present numerical model versus an analytical integrated solution. Concentration profile of aryl radical along y (at mid-height, i.e. z = 60 µm, and at mid-width, i.e. x = 0 µm) obtained by numerical simulation in the present study (full black line) and from an analytical solution (crosses) of the integrated reaction rate equation for aryl radical production. In this simulation, only the reaction of aryl radical production was considered, and NBD and KI were assumed to be well-mixed prior to the inlet of the main microfluidic channel and flowed at 100 µL/min (50 µL/min for each of the reactants). In such scenario, the residence times of the fluid elements at the middle of the channel cross-section (z = 60 µm, x = 0 µm) are similar due to the approximately flat velocity profile (**Supplementary Fig. 3a**), implying similar radical concentrations. Therefore, at that region, diffusion is negligible and the production of radical is the sole mechanism affecting the concentration of aryl radical. This implies that we can compare the concentration profile of aryl radical with the analytical solution of the reaction rate equation, obtained by integration.

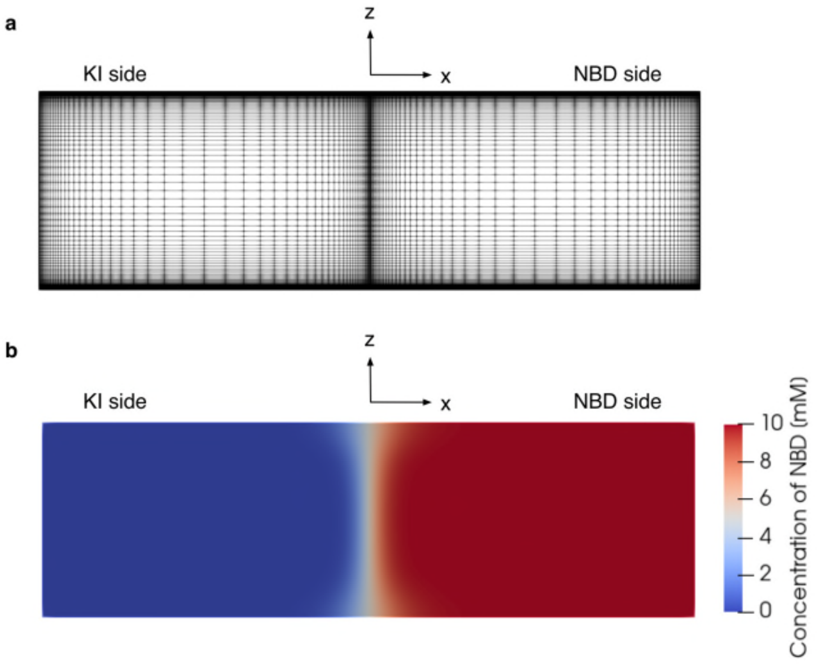

Supplementary Fig. 5. Numerical simulations. a, Cross-sectional image of xz-plane located in the middle of the main microchannel (*i.e.* at zone 2) showing the finely meshed domain. **b,** Concentration map of NBD in the xz-plane at the channel mid-length (*i.e.*, at zone 2).

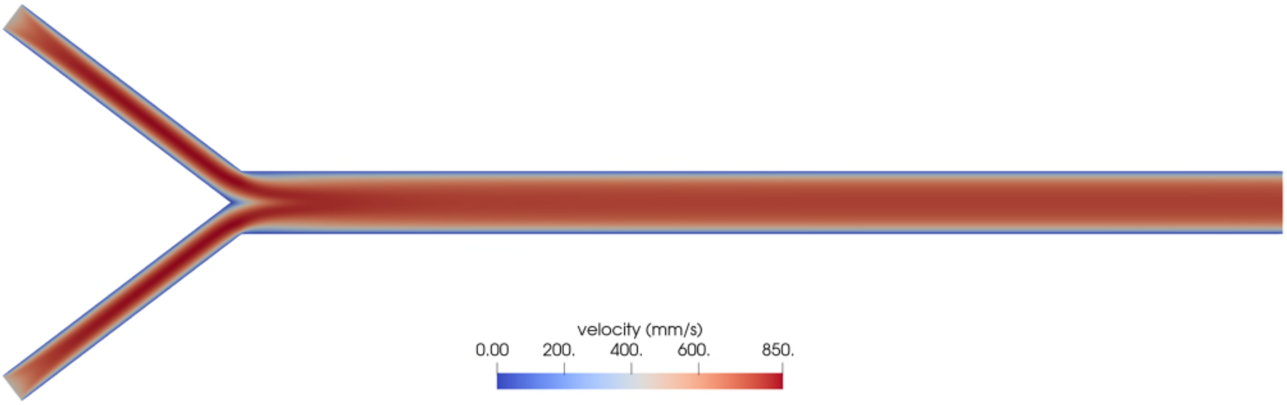

Supplementary Fig. 6. Velocity map of the flow in the microfluidic channel at mid-height (z = 60 µm) at 600 µL/min. The map shows that the flow becomes fully developed near the beginning of the main microfluidic chamber, meaning that the velocity profiles downstream that region do not change along the length of the chamber.

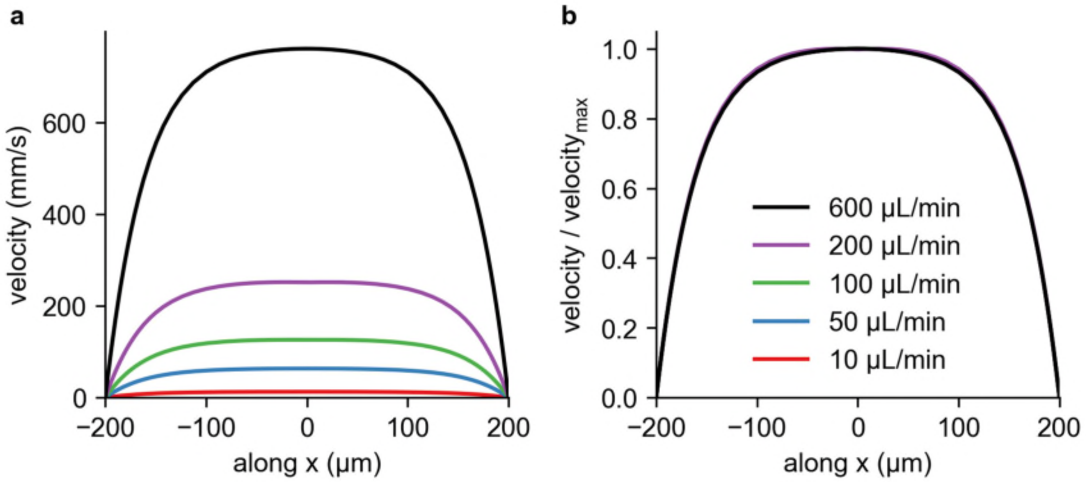

Supplementary Fig. 7. Velocity profile of the developed flow along the width of the main microfluidic channel (x-axis) at mid-height (z = 60 µm) for different flow rates. a-b, Velocity profile in zone 2 along x at mid-height of the channel, obtained from numerical simulations when the flow rate is varied from 10 to 600 µL/min. In panel **a,** the velocity is expressed in absolute value (mm/s). In panel **b**, the velocity is expressed in relative terms, by dividing the velocity by its maximum value for each flow rate (relative velocity varies from 0 to 1, where 1 corresponds to the maximum velocity).

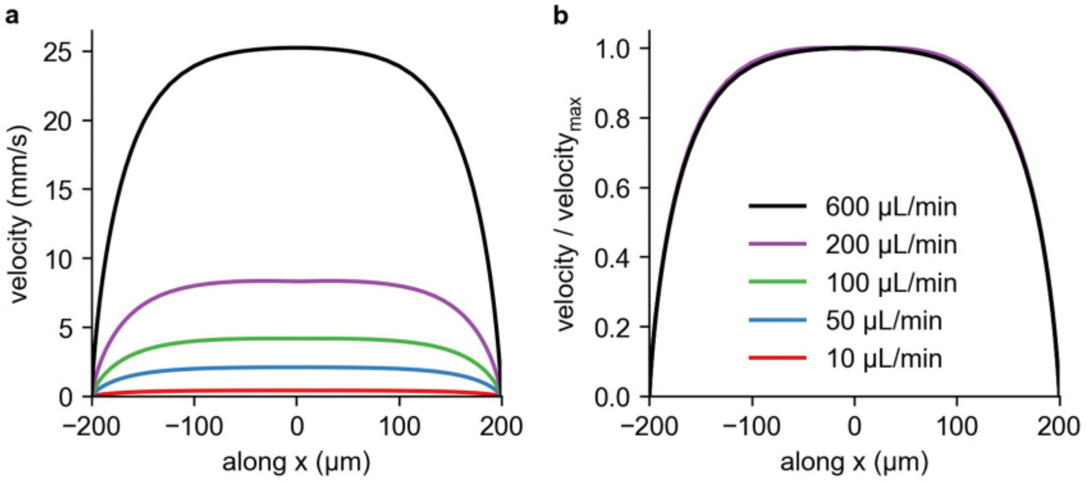

Supplementary Fig. 8. Velocity profile of the developed flow along the width of the main microfluidic channel (x-axis) near the HOPG substrate (z = 1 μm) for the different flow rates. a-b, Velocity profile in zone 2 along x near the substrate, obtained from numerical simulations when the flow rate is varied from 10 to 600 μL/min. In panel **a,** the velocity is expressed in absolute value (mm/s). In panel **b**, the velocity is expressed in relative terms, by dividing the velocity by its maximum value for each flow rate (relative velocity varies from 0 to 1, where 1 corresponds to the maximum velocity).

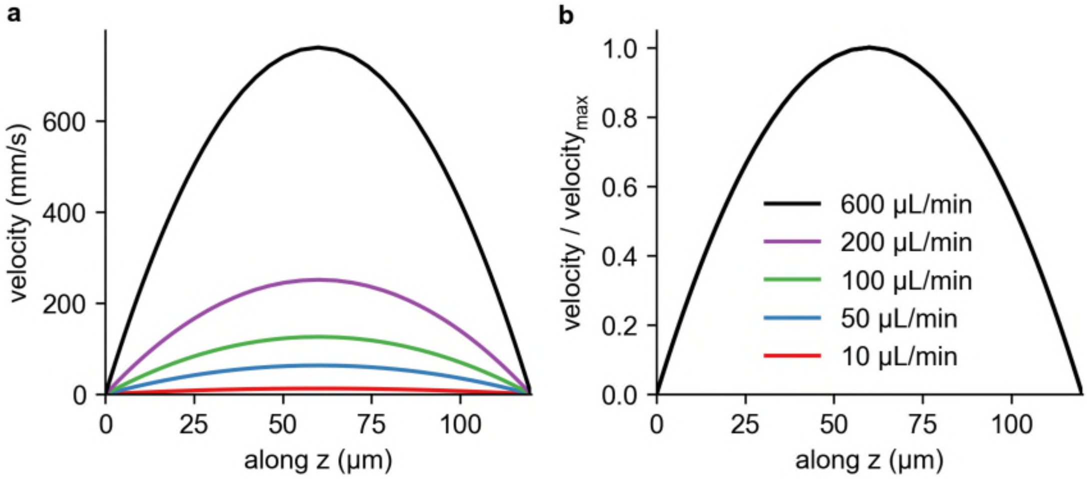

Supplementary Fig. 9. Velocity profile of the developed flow along the height of the main microfluidic channel (z-axis) at mid-width (x = 0 μm) for the different flow rates. a-b, Velocity profile in zone 2 along z at mid-width of the channel, obtained from numerical simulations when the flow rate is varied from 10 to 600 μL/min. In panel **a,** the velocity is expressed in absolute value (mm/s). In panel **b**, the velocity is expressed in relative terms, by dividing the velocity by its maximum value for each flow rate (relative velocity varies from 0 to 1, where 1 corresponds to the maximum velocity).

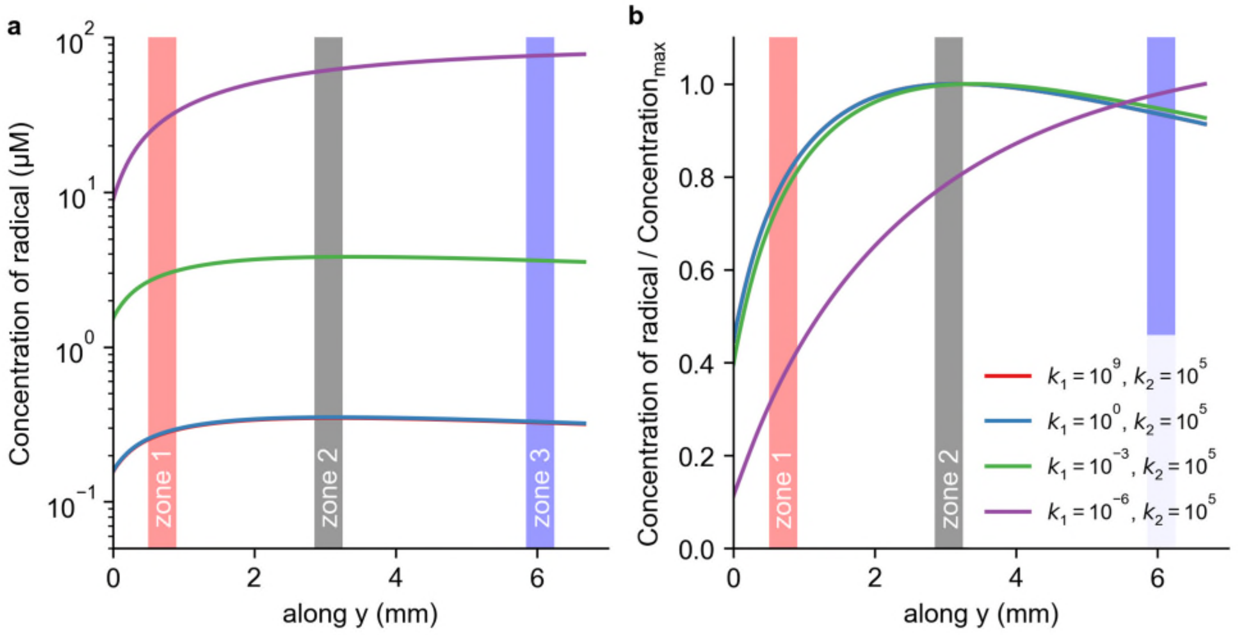

Supplementary Fig. 10. Concentration profiles of aryl radical along the main microfluidic chambers' length. a-b, The concentrations of aryl radical, obtained by numerical simulation for a flow rate of 50 µL/min and considering different values of k_1 for $k_2 = 10^5$, are plotted along the length of the main microchannel (along y) in the middle of its width (x = 0 µm) in close proximity to the HOPG (z = 0 µm). In panel **a,** the concentration of aryl radical is expressed in absolute value (µM). In panel **b**, the concentration of aryl radical is expressed in relative terms, by dividing the concentration by its maximum value in each curve (relative concentration varies from 0 to 1, where 1 corresponds to the maximum concentration). The values of k_1 and k_2 are given in $m \cdot s^{-1}$ and $M^{-1} \cdot s^{-1}$, respectively.

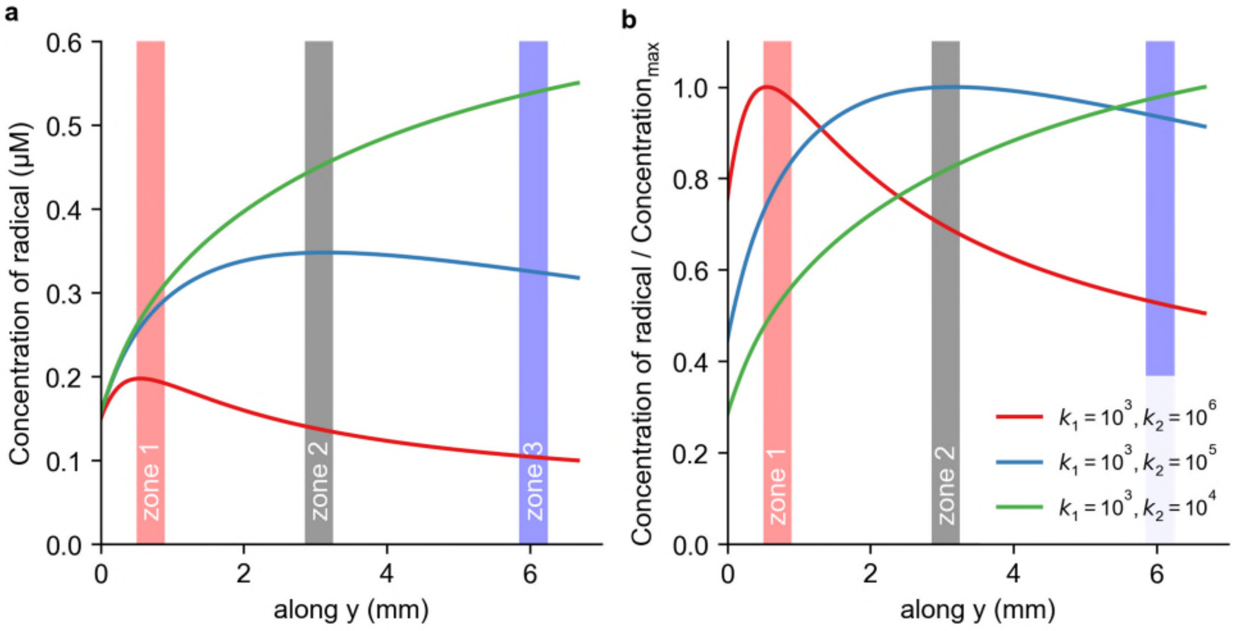

Supplementary Fig. 11. Concentration profiles of aryl radical along the main microfluidic chambers' length. a-b, The concentrations of aryl radical, obtained by numerical simulation for a flow rate of 50 µL/min and considering $k_1 = 10^3$ and different values of k_2, are plotted along the length of the main microchannel (along y) in the middle of its width (x = 0 µm) in close proximity to the HOPG (z = 0 µm). In panel **a,** the concentration of aryl radical is expressed in absolute value (µM). In panel **b**, the concentration of aryl radical is expressed in relative terms, by dividing the concentration by its maximum value in each curve (relative concentration varies from 0 to 1, where 1 corresponds to the maximum concentration). The values of k_1 and k_2 are given in $m \cdot s^{-1}$ and $M^{-1} \cdot s^{-1}$, respectively.

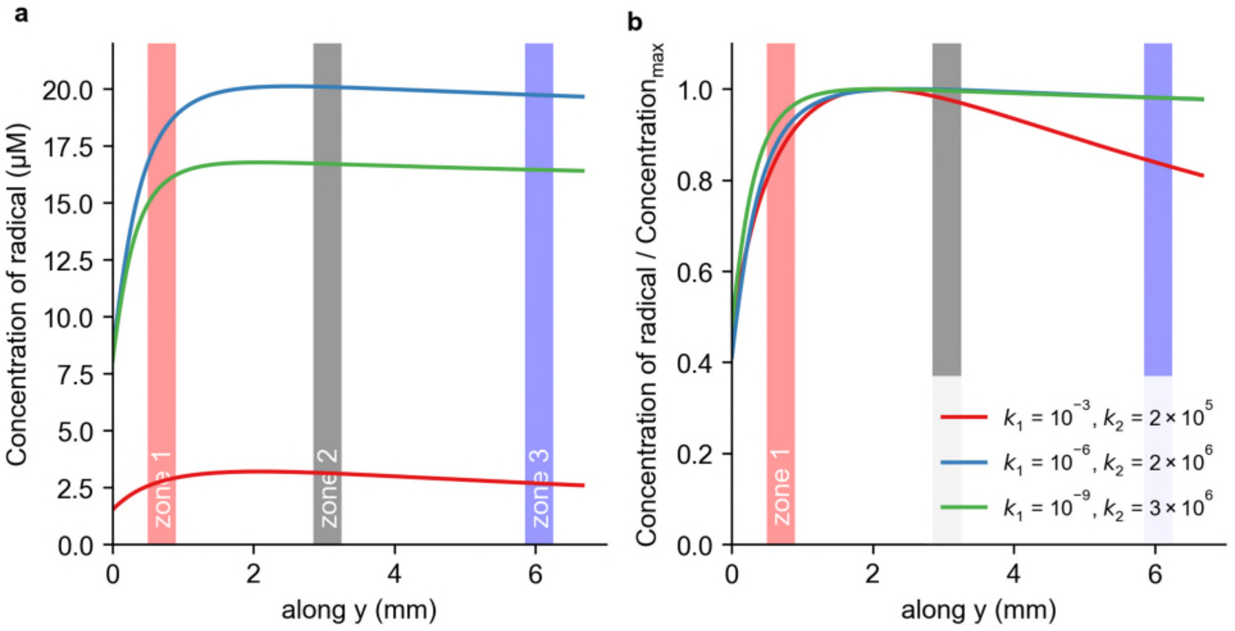

Supplementary Fig. 12. Concentration profiles of aryl radical along the main microfluidic chambers' length. a-b, The concentrations of aryl radical, obtained by numerical simulation for a flow rate of 50 µL/min and considering different k_1 and different k_2, are plotted along the length of the main microchannel (along y) in the middle of its width (x = 0 µm) in close proximity to the HOPG (z = 0 µm). In panel **a,** the concentration of aryl radical is expressed in absolute value (µM). In panel **b**, the concentration of aryl radical is expressed in relative terms, by dividing the concentration by its maximum value in each curve (relative concentration varies from 0 to 1, where 1 corresponds to the maximum concentration). The values of k_1 and k_2 are given in $m \cdot s^{-1}$ and $M^{-1} \cdot s^{-1}$, respectively.

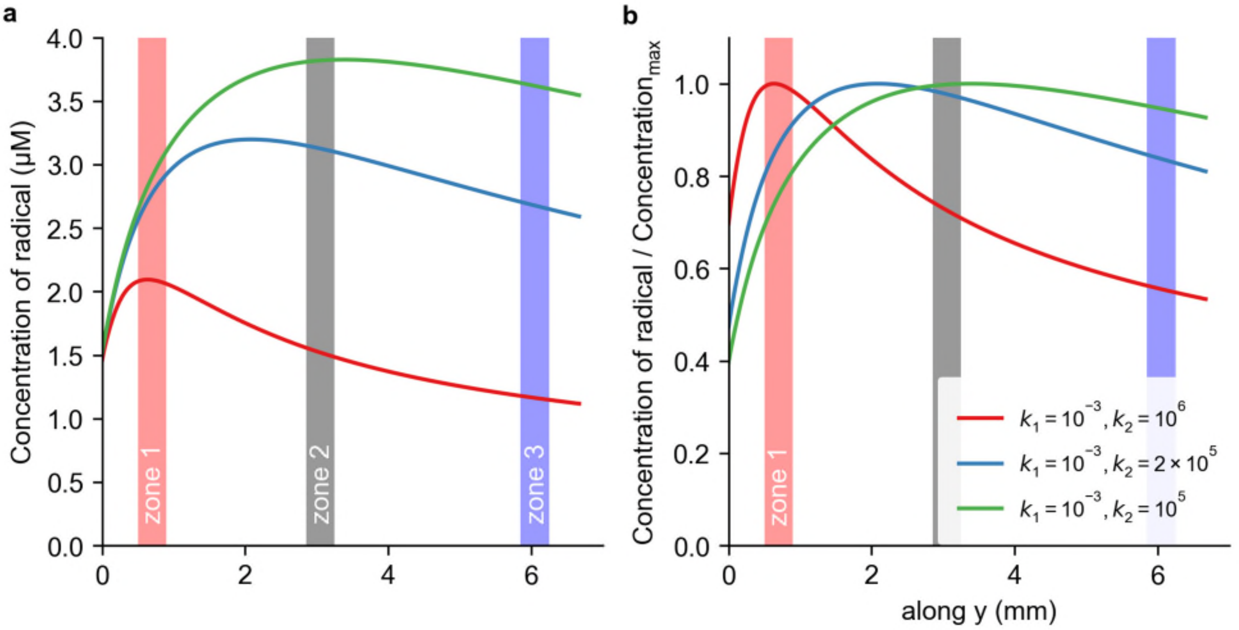

Supplementary Fig. 13. Concentration profiles of aryl radical along the main microfluidic chambers' length. a-b, The concentrations of aryl radical, obtained by numerical simulation for a flow rate of 50 µL/min and considering $k_1 = 10^{-3}$ and different k_2, are plotted along the length of the main microchannel (along y) in the middle of its width (x = 0 µm) in close proximity to the HOPG (z = 0 µm). In panel **a,** the concentration of aryl radical is expressed in absolute value (µM). In panel **b**, the concentration of aryl radical is expressed in relative terms, by dividing the concentration by its maximum value in each curve (relative concentration varies from 0 to 1, where 1 corresponds to the maximum concentration). The values of k_1 and k_2 are given in $m \cdot s^{-1}$ and $M^{-1} \cdot s^{-1}$, respectively.

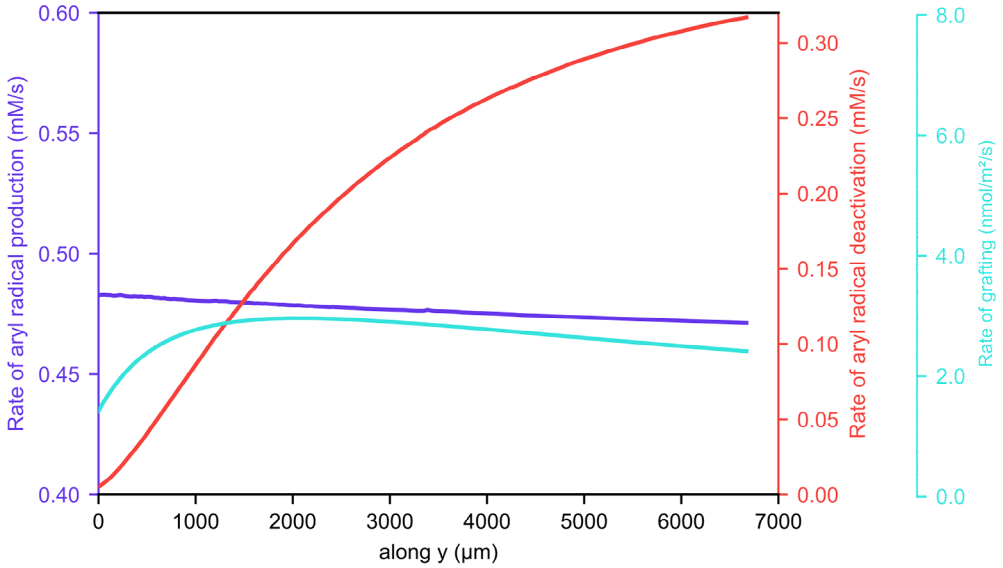

Supplementary Fig. 14. Rate of aryl radical production, deactivation and grafting along the length of the main microchannel. The reaction rates of aryl radical production (purple blue), deactivation (red) and grafting onto HOPG (cyan), obtained by numerical simulation, are plotted along the length of the main microchannel (along y) in the middle of its width (x = 0 µm) in close proximity to the HOPG (z = 0 µm). The flow rate of each reactant stream was 50 µL/min. The reaction rates shown here were calculated based on equations of aryl radical production, deactivation and grafting, described in **the numerical simulation methods**, considering $k_1 = 10^{-3}$ m·s^{-1} and $k_2 = 2 \times 10^5$ M^{-1}·s^{-1}.

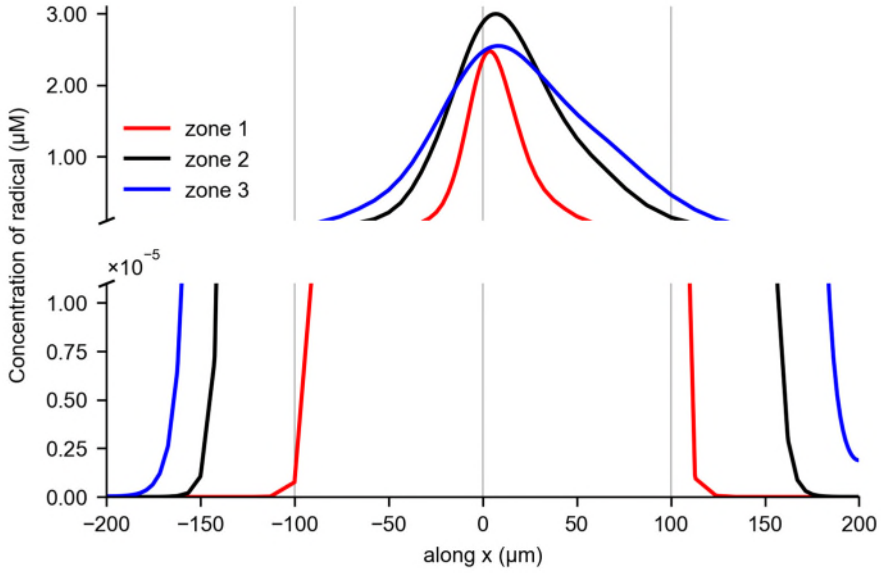

Supplementary Fig. 15. Predicted concentration of aryl radicals near the HOPG substrate along the main microfluidic channel's width (x-axis). Three different zones (zone 1-3) along the length of the main microfluidic channel are represented respectively in red, black and blue. The break in the concentration axis is used to clearly show that there are still aryl radicals available at 200-µm far away from the center of the main microfluidic channel (*i.e.* x = 0). The numerical results show that the aryl radicals can cover the entire width of the main microfluidic channel. Concentration curves calculated based on equations of aryl radical production, deactivation and grafting, described in **the numerical simulation methods**, considering $k_1 = 10^{-3}$ m·s^{-1} and $k_2 = 2 \times 10^5$ M^{-1}·s^{-1}.

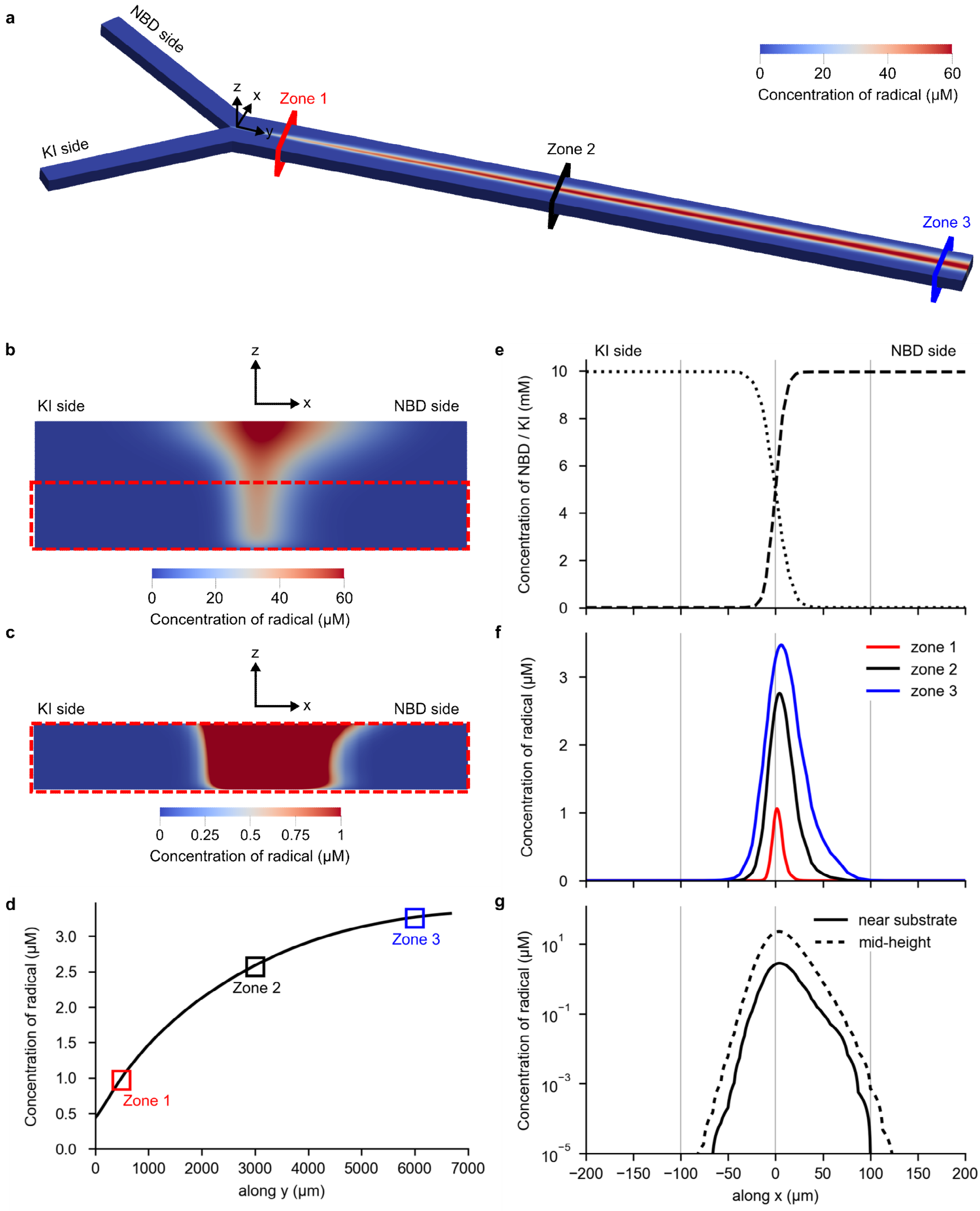

Supplementary Fig. 16. Numerical results of the mass transport in the microfluidic device for a hypothetical case considering free-slip boundary condition at the HOPG substrate. a, Concentration map of the aryl radical concentrations in the entire main microfluidic channel. **b,** Concentration map of aryl radical at the channel mid-length (i.e. xz-plane, zone 2). **c,** Concentration map of aryl radical at the channel mid-length (i.e. xz-plane, zone 2), considering a very narrow concentration range to show the aryl radical concentration close to the HOPG substrate. **d,** Concentration of aryl radical at the HOPG substrate along the main microfluidic channel length (along y-axis, x=0). **e,** Concentration profiles of the reactants (KI and NBD) along the width of the channel (along x-axis, zone 2) near the HOPG substrate. **f,** Concentration profiles of aryl radical near the HOPG substrate for different positions along the channel length (zone 1 [near inlet], zone 2 [mid-length] and zone 3 [near outlet]). **g**, Concentration profiles (in log scale) of aryl radical near the HOPG substrate and at the mid-height of the channel (zone 2; the curves are not smooth due to the logarithmic scale used in the vertical y-axis). Representative coordinate systems and color codes for different channel zones are given in panel **a**.

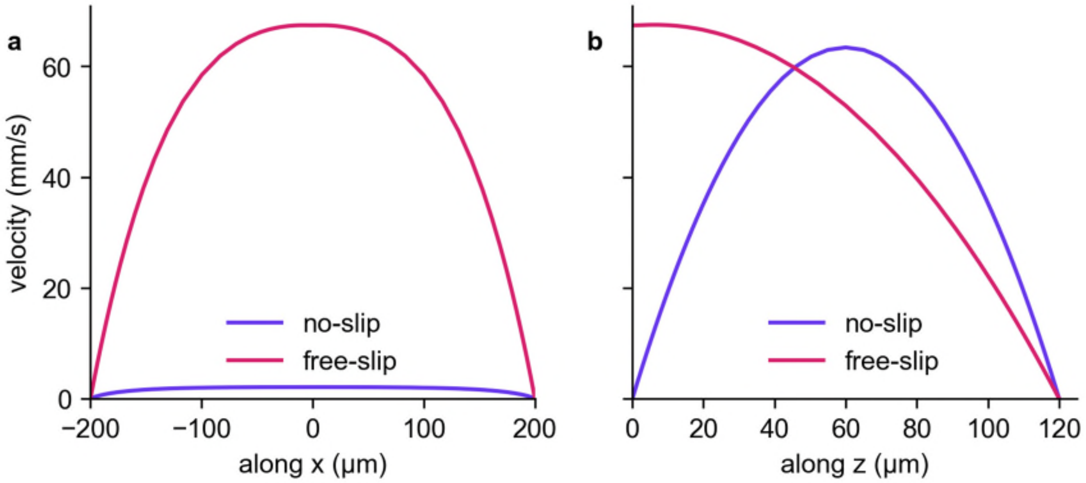

Supplementary Fig. 17. Velocity profile of the developed flow in the main microfluidic channel for a flow rate of 50 µL/min considering no-slip and free-slip boundary conditions in numerical simulations. a, Velocity profile in zone 2 along the width of the main microfluidic channel (x-axis) near the HOPG substrate (z = 1 µm). **b,** Velocity profile in zone 2 along the height of the main microfluidic channel (z-axis) in the middle of its width (x = 0 µm).

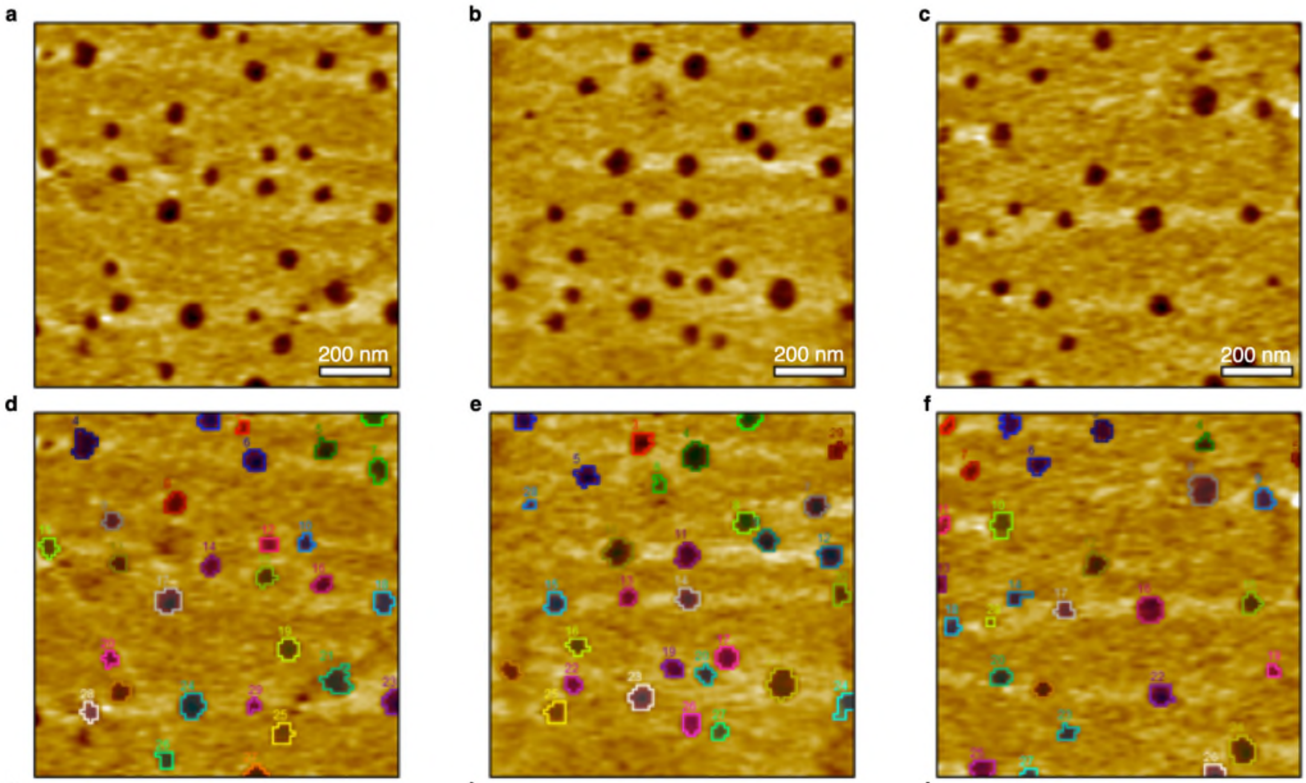

g

NO.	Area/nm²	Diameter/nm
1	2491.3	56.3
2	2076.1	51.4
3	968.9	35.1
4	3875.4	70.2
5	2629.7	57.9
6	3045	62.3
7	2629.7	57.9
8	2768.1	59.4
9	2076.1	51.4
10	1660.9	46
11	2076.1	51.4
12	1660.9	46
13	1937.7	49.7
14	1937.7	49.7
15	2352.9	54.7
16	2076.1	51.4
17	4152.2	72.7
18	2906.6	60.8
19	2906.6	60.8
20	1384.1	42
21	4567.4	76.3
22	2906.6	60.8
23	2629.7	57.9
24	4013.8	71.5
25	2768.1	59.4
26	1799.3	47.9
27	1660.9	46
28	1799.3	47.9
29	1107.3	37.5

h

NO.	Area/nm²	Diameter/nm
1	2352.9	54.7
2	2629.7	57.9
3	2906.6	60.8
4	4567.4	76.3
5	2629.7	57.9
6	1384.1	42
7	3183.4	63.7
8	3321.8	65
9	2629.7	57.9
10	4844.3	78.5
11	3321.8	65
12	3460.2	66.4
13	1799.3	47.9
14	3045	62.3
15	2629.7	57.9
16	2214.5	53.1
17	2906.6	60.8
18	2768.1	59.4
19	2214.5	53.1
20	2076.1	51.4
21	5674.7	85
22	1799.3	47.9
23	4013.8	71.5
24	2768.1	59.4
25	2768.1	59.4
26	2491.3	56.3
27	1799.3	47.9
28	692	29.7
29	1522.5	44
30	2214.5	53.1

i

NO.	Area/nm²	Diameter/nm
1	2768.1	59.4
2	1107.3	37.5
3	2629.7	57.9
4	1522.5	44
5	692	29.7
6	2214.5	53.1
7	1522.5	44
8	5951.5	87
9	2629.7	57.9
10	3321.8	65
11	1384.1	42
12	3321.8	65
13	1107.3	37.5
14	1660.9	46
15	2491.3	56.3
16	4429	75.1
17	2076.1	51.4
18	1799.3	47.9
19	1107.3	37.5
20	2352.9	54.7
21	2214.5	53.1
22	3321.8	65
23	1799.3	47.9
24	4705.8	77.4
25	2906.6	60.8
26	1937.7	49.7
27	968.9	35.1
28	553.6	26.5

Supplementary Fig. 18. AFM image analysis. For a specific location in the main microfluidic channel, an overview AFM image (6 µm by 6 µm) was obtained for evaluating the general morphology of this position. **a-c,** To analyze the features of the functionalized surface in more detail, 3 magnified AFM images (1 µm by 1 µm) were obtained. **d-f,** The same AFM images marked with circles for the corral (empty) areas and **g-i,** the corresponding statistics obtained from the marked AFM images, including the number (NO.), area and diameter of corrals. For each position in the main microfluidic channel, three images were used to calculate the average value and standard deviation. AFM images are obtained from the sample prepared with a flow rate of 50 µL/min and a flow time of 5 minutes. Scale bars: 200 nm.

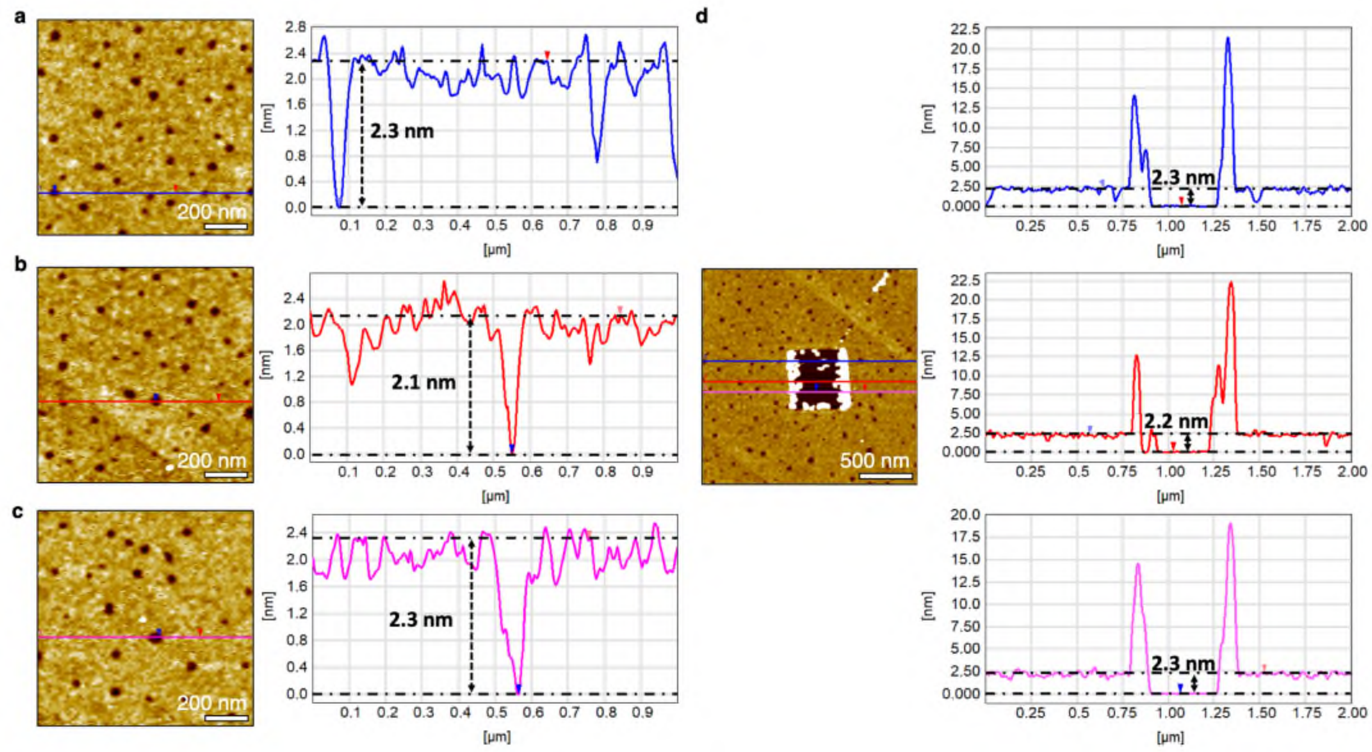

Supplementary Fig. 19. Confirmation of the layer thickness by a scratching test. a-c, Three AFM images and the corresponding line profiles (respectively in blue, red and pink) used to determine the layer thickness based on the corrals in the functionalized layer (2.2 ± 0.1 nm). **d,** An AFM image and three-line profiles (respectively in blue, red and pink) obtained after a scratching test. The height of the layer was 2.3 ± 0.1 nm. The thicknesses obtained from the two methods show very similar values. AFM images are obtained from a sample prepared with a flow rate of 50 µL/min and a flow time of 60 minutes. Scale bars: 200 nm in **a-c**, and 500 nm in **d**.

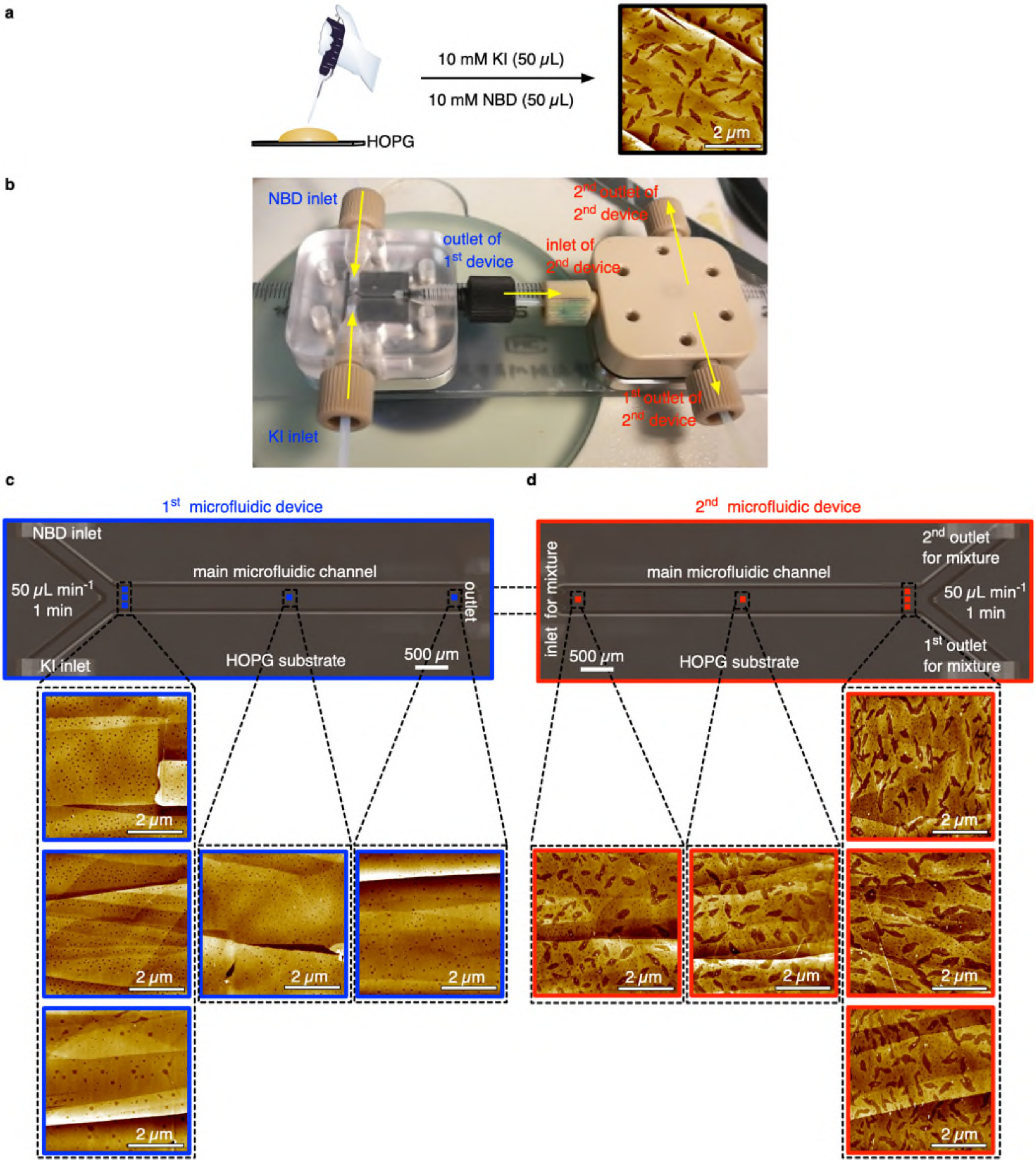

Supplementary Fig. 20. Comparison of the different approaches for the covalent modification of HOPG. a, The drop casting approach: 50 µL NBD (10 mM) and 50 µL KI (10 mM) were premixed and dropped casted onto the HOPG for 10 minutes. **b,** Microfluidic approach: the samples were prepared with two microfluidic devices connected in series (50 µL/min and 1 minute). The first microfluidic device was connected to the second one such that the outlet of first device is the inlet for second device. **c-d,** AFM images obtained from different locations along the main microfluidic channel show that in the first microfluidic device a spatially controlled functionalization of the HOPG is possible **c,** while in **d,** the AFM images obtained in the second microfluidic device indicate that the functionalization of the HOPG in this case is not controlled. The AFM images shown in **d** are very similar to the AFM images obtained from the bulk experiments, *i.e.* the drop-casting method.

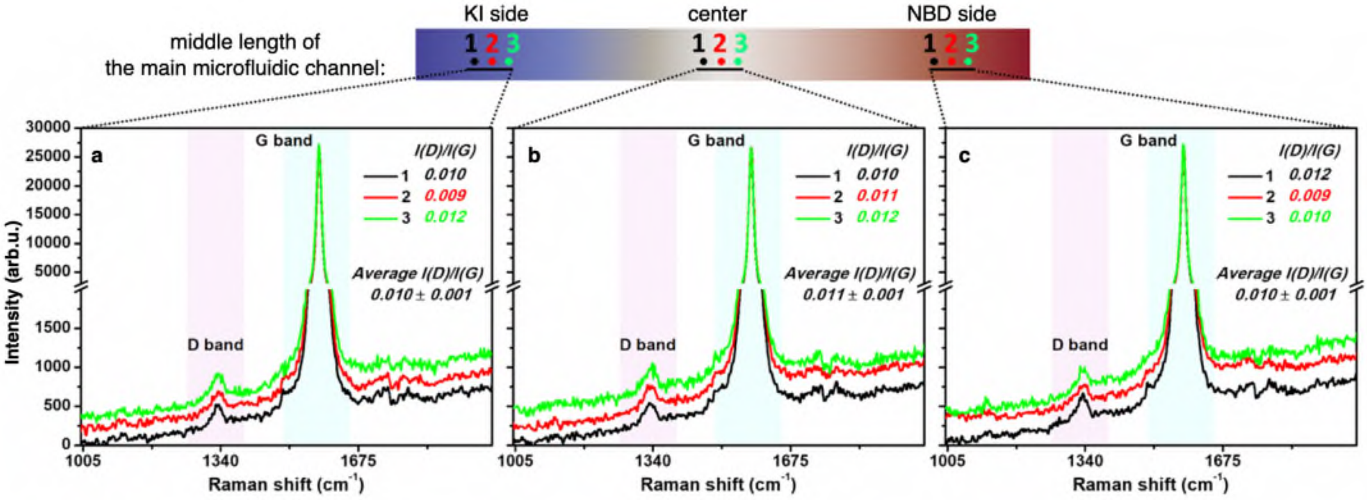

Supplementary Fig. 21. Raman spectra obtained at different locations and along the mid-length of the channel (zone 2). a-c, Raman spectra were taken from different locations (*i.e* **a,** KI side, **b,** center and **c,** NBD side) at zone 2 for the sample with 50 µL/min and 5 minutes flow speed and time. From each location three Raman spectra were taken, separated by about 6 µm. The ratio of I(*D*)/I(*G*) was determined for each spectrum and the average I(*D*)/I(*G*) ratio and standard deviation calculated for each location. The Raman spectra from the KI side, center and NBD side (from left to right) of the channel are very similar in appearance and I(*D*)/I(*G*) ratio.

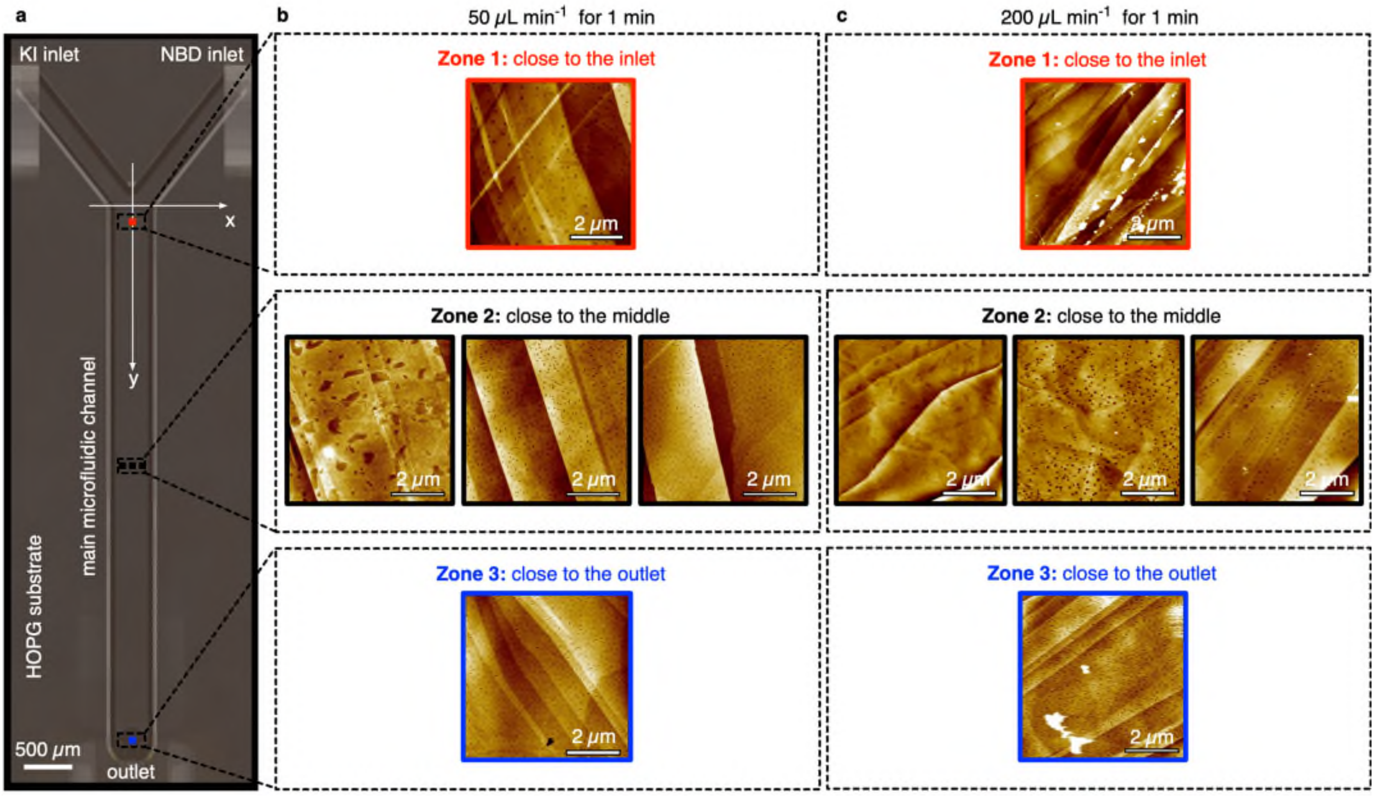

Supplementary Fig. 22. Tailoring RD zones by changing flow rates. a, Top view of the microfluidic network showing the inlets and outlet ports as well as the representative locations where the AFM images were acquired. Panel **a** also shows the coordinate system used in our studies. **b-c,** AFM images of the samples prepared with a flow rate of 50 µL/min and 200 µL/min (1 minute), respectively. The AFM images are color coded with respect to the locations from where they were acquired along the main microfluidic channel (red, black and blue strokes respectively for zone 1, zone 2 and zone 3).

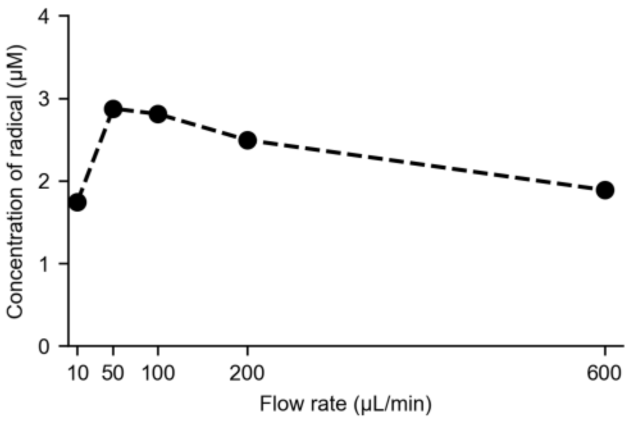

Supplementary Fig. 23. Concentration of aryl radical at the center (x = 0) of zone 2 for the different flow rates. The graph shows the change in aryl radical concentration when the flow rate is varied from 10 to 600 μL/min due to the complex dynamics between production and deactivation rates. The concentrations values were calculated based on equations of aryl radical production, deactivation and grafting, described in **the numerical simulation methods**, considering $k_1 = 10^{-3}$ m·s^{-1} and $k_2 = 2 \times 10^5$ M^{-1}·s^{-1}.

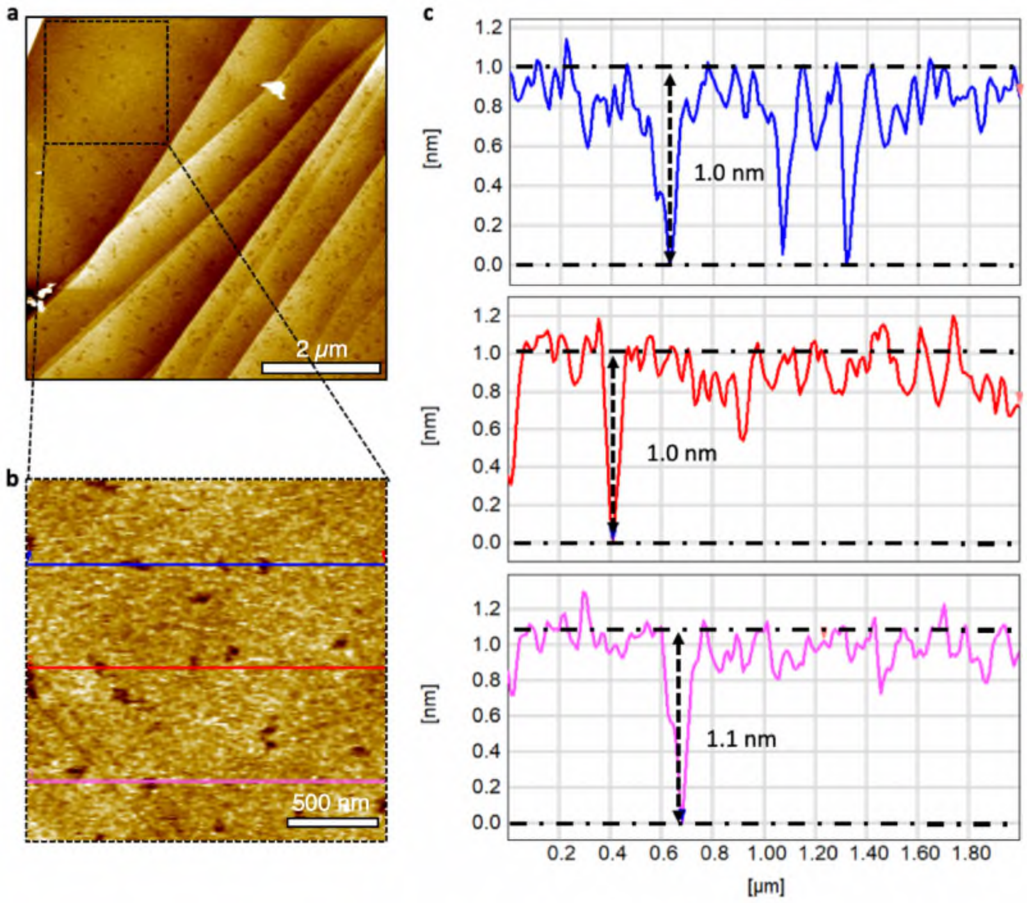

Supplementary Fig. 24. Height analysis of the sample prepared with a flow rate of 10 μL/min (flow time of 10 minutes). a, The AFM image obtained from the middle of the main microfluidic channel (at the location represented in **Fig. 5**) and **b,** its magnified version with three lines to obtain three height profiles. The height profiles are shown in blue, red and pink. **c,** Height profiles corresponding to the blue, red and pink lines shown in **b** (respectively from top to bottom). This analysis reveals an average thickness of 1.0 ± 0.1 nm. Scale bars: 2 μm in **a**, and 500 nm in **b**.

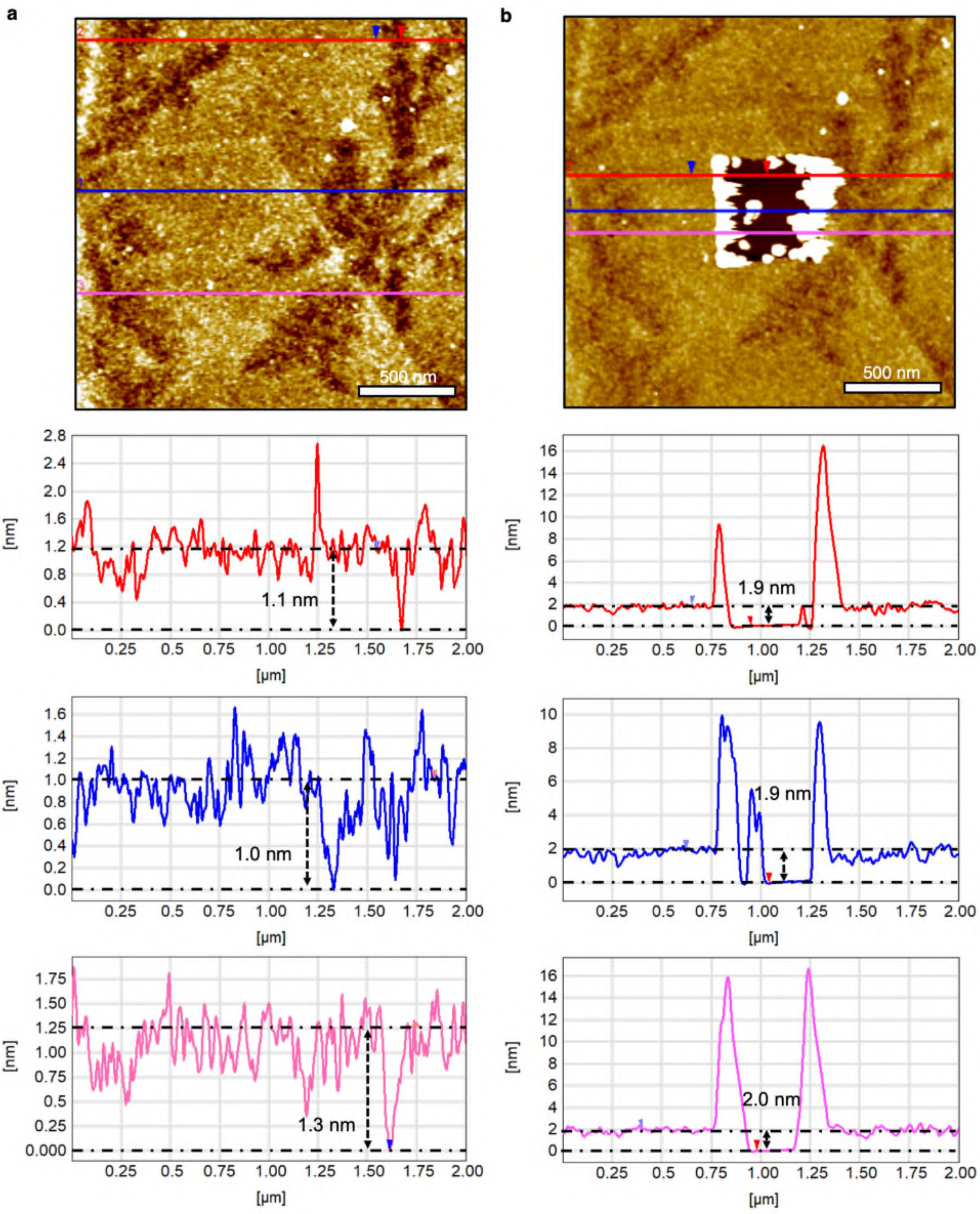

Supplementary Fig. 25. Height analysis of the sample prepared with a flow rate of 10 µL/min (flow time of 60 minutes). a-b, AFM images obtained from the middle of the main microfluidic channel (at the location represented in **Fig. 5**) with their respective line profiles. In panel **a,** the measured average thickness is 1.1 ± 0.2. However, in this case no clear corral formation is apparent. **b,** Accordingly, a scratching test is performed and an average thickness of 1.9 ± 0.1 is measured. Note that both analyses result in a different layer thickness since at this flow rate (10 µL/min), the second layer grows on a highly dense monolayer. Scale bars: 500 nm.

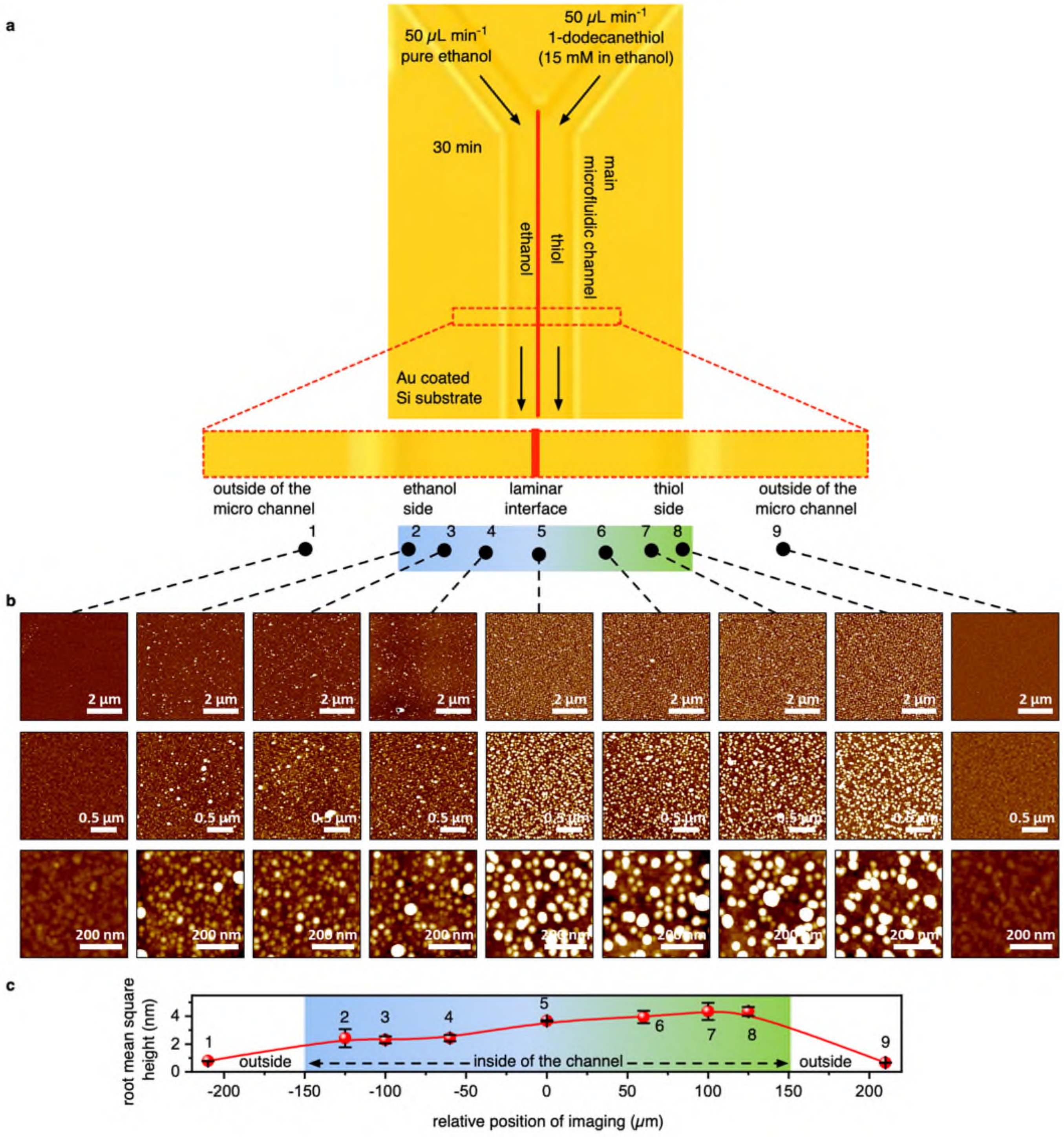

Supplementary Fig. 26. Gradient formation on Au-coated Si. a, Schematic drawing of PDMS-based microfluidic device and experimental configuration. **b,** AFM images showing the gradient of 1-dodecanethiol over Si-Au substrate. From left to right: position (1), outside of the main microfluidic channel (left side); positions from (2) to (8), locations measured along the width of the main microfluidic channel (300 μm) and from the pure EtOH-laden flow towards the 1-dodecanethiol-laden flow. Note that the concentration of 1-dodecanethiol increases when moving towards the 1-dodecanethiol-laden flow; and position (9) outside of the main microfluidic channel (right side). Scale bars: 2 μm, 0.5 μm, 0.2 μm for top, middle and bottom rows, respectively. **c,** Root mean square height calculated from AFM images with respect to relative position for quantification of gradient formation under the main channel area. The error bars correspond to the standard deviation of the 3 measurements around corresponding positions.

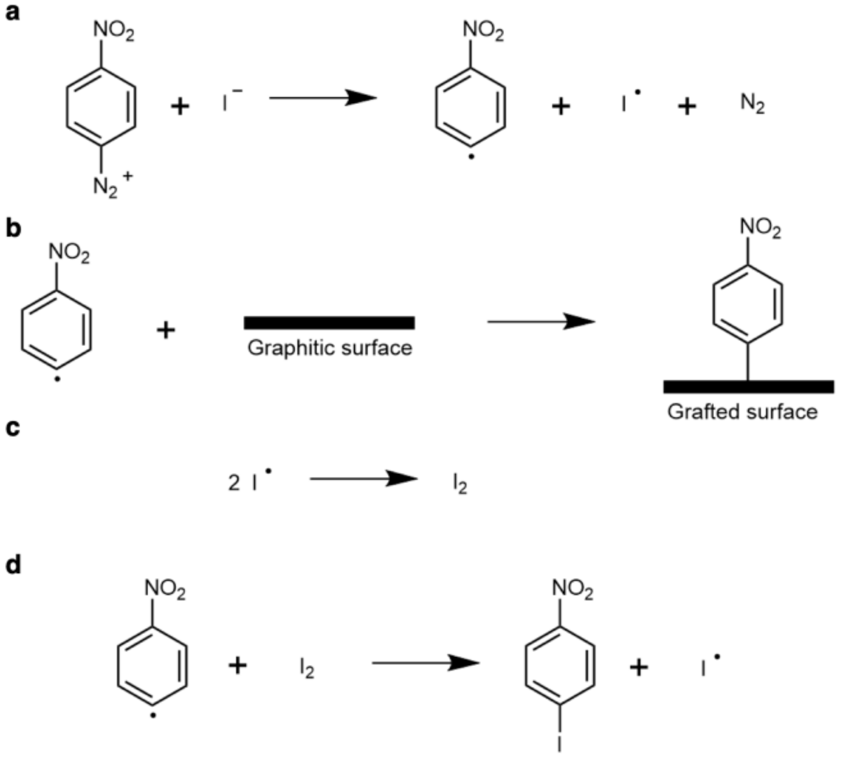

Supplementary Fig. 27. Reaction mechanism affecting the concentration of aryl radicals within the microfluidic channel. a, The production of the aryl radical. **b,** The consumption of aryl radical by the surface attachment (grafting). **c,** The formation of iodine (I_2). **d,** The deactivation of the aryl radical with I_2.

Supplementary Tables

Supplementary Table 1. Combinations of reaction rate constants k_1 and k_2 that were tested to identify those implying aryl radical concentration profiles (in the numerical simulations) that are consistent with the experimentally-observed layer thicknesses. * identifies the combinations that reasonably fit the experimental data, and *** highlights the combination that was the best fit to the experimental data and that was used in the main numerical simulations shown in this work.

**Combination**	**k_1 ($m\cdot s^{-1}$)**	**k_2 ($M^{-1}\cdot s^{-1}$)**
1	10^{-9}	$2x10^6$
2*	10^{-9}	$3x10^6$
3	10^{-9}	$4x10^6$
4	10^{-9}	$7x10^6$
5	10^{-9}	$1x10^7$
6	10^{-6}	$1x10^5$
7	10^{-6}	$5x10^5$
8	10^{-6}	$7x10^5$
9	10^{-6}	$1x10^6$
10*	10^{-6}	$2x10^6$
11	10^{-6}	$5x10^6$
12	10^{-6}	$1x10^7$
13	10^{-6}	$1x10^8$
14*	10^{-3}	$1x10^5$
15***	10^{-3}	$2x10^5$
16	10^{-3}	$3x10^5$
17	10^{-3}	$1x10^6$
18	10^{-3}	$1x10^7$
19	10^{-3}	$1x10^8$
20*	10^0	$1x10^5$
21	10^0	$1x10^6$
22	10^0	$1x10^8$
23	10^3	$1x10^4$
24*	10^3	$1x10^5$
25	10^3	$1x10^6$
26	10^3	$1x10^8$
27*	10^6	$1x10^5$
28*	10^9	$1x10^5$

Supplementary Table 2. The grafting layer thickness, size and density of corrals in the center of the zone 2 as the function of the flow time (min) and flow rate (µL/min). Columns **a**, **b**, and **c** show average layer thickness (in nm), average diameter of corrals (in nm), and average number of corrals per square micrometers, respectively.

**Flow rate**	10 µL/min			50 µL/min			100 µL/min			200 µL/min			600 µL/min		
**Flow time**	**a**	**b**	**c**	**a**	**b**	**c**	**a**	**b**	**c**	**a**	**b**	**c**	**a**	**b**	**c**
1 min				1.5	60.8	30.3	1.3	72.6	7.7	1.2	82.5	12.3			
5 min				2.0	55.2	29.0									
10 min	1.0	61.9	13.7	2.1	49.7	24.7							0.9	42.8	53.0
60 min	2.0			2.2	41.3	31.7									
120 min				3.0	46.3	9.3									

Supplementary Notes

Supplementary Note 1: Calculation of diffusion coefficient with DOSY for numerical simulations

The diffusion coefficients were identified by fitting the diffusion profile (the normalized signal intensity as a function of the gradient strength G) at the chemical shift of each signal in the DOSY spectrum with an exponential function using the variant of Stejskal–Tanner equation adapted to the particular pulse sequence used[4].

Supplementary Note 2: Reaction mechanism

The reaction mechanism affecting the concentration of aryl radicals within the microfluidic channel includes four different main reactions, which involve the formation of the aryl radical and its consumption in the grafting processes, as wells as side reactions leading to the deactivation of the aryl radical[5]. The formation of the aryl radicals occurs when NBD is reduced in a 1-electron process with iodide, resulting in the homolytic breakage of the C-N bond to produce the aryl radical, as well as iodine radicals and N_2 gas as by-products. (**Supplementary Fig. 27a**) The aryl radical formed in the previous step can react with a double bond of the graphitic surface to form the grafted layer. (**Supplementary Fig. 27b**) Simultaneously, the iodine radicals generated in the first step are highly reactive and can recombine to form molecular iodine. (**Supplementary Fig. 27c**) The molecular iodine is a well-known scavenger of aryl radicals[6] and reacts rapidly with them to form aryl iodide. (**Supplementary Fig. 27d**) Since aryl iodide is no longer reactive, the latter step effectively deactivates the radical, as it eliminates its capacity to react with the substrate to form the grafted layer. Therefore, if enough iodine builds up, this reaction will compete with the grafting process and it will be inhibited.

Supplementary Note 3: Calculation of reaction kinetics with UV-Vis spectroscopy for numerical simulations

The method of initial rates was used to determine the rate law of aryl radical formation from NBD and KI to be used in the numerical simulations. The decrease of concentration of NBD was monitored by its absorbance at 260 nm.

A set of reactions was performed with a fixed concentration of KI of 175 μM and NBD concentrations ranging from 25 to 95 μM. The initial rates of these reactions were fitted to an equation of the form: $\log(v) = \log(k_1) + n \cdot \log([NBD])$, where v is the initial reaction rate, k_1 the apparent rate constant, n the reaction order on NBD, and $[NBD]$ the concentration of NBD. With this procedure, a reaction order on NBD concentration of 0.62 was obtained.

A second set of reactions was performed with a fixed concentration of NBD of 25 μM and KI concentrations ranging from 95 to 120 μM. The initial rates of these reactions were fitted to an equation of the form: $\log(v) = \log(k_2) + m \cdot \log([KI])$, where v is

the initial reaction rate, k_2 the apparent rate constant, *m* the reaction order on KI, and [*KI*] the concentration of KI. This resulted in a reaction order on KI concentration of 0.43.

The rate constant k can be derived from the apparent rate constants once the reaction orders of each reactant are known, yielding a value of 0.13 s^{-1} $M^{-0.05}$.

Therefore, the proposed rate equation for the aryl radical formation reaction is:

$$v = 0.13 \cdot [NBD]^{0.62} \cdot [KI]^{0.43}$$

Supplementary Note 4: Analysis of flow in the main microfluidic chamber for flow rates of 10 to 600 µL/min

The flow development length in the main microfluidic chamber, i.e. the length of channel needed for the flow to fully develop, grows with the Reynolds number of the flow[7]. Given the low Reynolds number of the flows for all the conditions tested (ranging from 1 to 77 for 10 µL/min and 600 µL/min, respectively), the corresponding development lengths are small and, because of that, the flow becomes fully developed near the beginning of the main microfluidic chamber (around zone 1), even for the highest flow rate tested (600 µL/min; **Supplementary Fig. 6**). As expected, the velocity of the developed flow along the width of the main microfluidic chamber at mid-height (z = 60 µm) increases significantly with increasing flow rate (**Supplementary Fig. 7a**). Moreover, and as expected, the velocity profiles obtained for different flow rates have similar shapes when represented as dimensionless velocity profiles (obtained by dividing each profile by its maximum velocity; **Supplementary Fig. 7b**). A higher velocity implies a lower residence time during which the reactants can diffuse and react with the HOPG substrate. Importantly, the similar dimensionless velocity profiles for the different flow rates indicate that the velocity of the fluid increases proportionally across the width of the channel, therefore implying proportionally smaller residence times across the width. This indicates that the change in flow rate offers a way to precisely control the residence times, and thus the chemical gradients across the entire width of the microfluidic reactor – key aspects to control the grafting process and the resulting layer formation.

The relation between flow rate and velocity/residence time is similar when the fluid is near the HOPG substrate (z = 1 µm) both in absolute (**Supplementary Fig. 8a**) and in relative (**Supplementary Fig. 8b**) terms. However, the absolute velocities were much smaller near the substrate (z = 1 µm) than in the middle of the channel height (z = 60 µm). This is due to the no-slip condition prevailing at the substrate (and all the other surfaces of the microfluidic device) which reduces the velocity of the fluid elements in its vicinity (**Supplementary Fig. 9a-b**). The no-slip condition is a good approximation of the behavior of most viscous flows near walls, in which the adherence forces between the substrate and the fluid molecules that are closest to it are greater than the cohesion forces between fluid molecules. Because of the no-slip condition, fluid elements in contact with the substrate (and all

other surfaces) will have null velocity, and those further away from the substrate/walls will have progressively larger velocities (**Supplementary Figs. 9a-b**). Importantly, because the velocity of the flow near the HOPG substrate is much smaller than that in the middle of the channel height (z = 60 μm), the residence times will be much larger, implying larger RD zones. Furthermore, because the height of the device (120 μm, z direction) is much smaller than its width (400 μm, x direction), the no-slip condition prevailing at the upper and lower surfaces of the microfluidic device leads to velocity profiles along the width (x direction) that are flatter in the central region of the channel (**Supplementary Figs. 8a-b**). As a consequence, the residence times vary little in that region of the channel, which is particularly convenient to control the reaction time. This shows that the no-slip condition prevailing at the device surfaces is critical to control the substrate functionalization, from the reaction times in the RD zone, to the exact width of substrate to be functionalized.

Supplementary Note 5: Screening of reaction rate constants that fit the experimental data of layer thicknesses

The aryl radical formation, deactivation and grafting processes were modeled based on the equations described in the **numerical simulation methods in the main text.** Since the rate constants of the grafting reaction (k_1) and of the I_2 formation (k_2) are unknown, we ran simulations for 28 different combinations of k_1 and k_2 (**Supplementary Table 1**) to investigate their influence over the aryl radical concentration inside the main microfluidic chamber, and to identify the values fitting best the experimental data of layer thickness.

The value of k_1 dictates how fast the grafting reaction occurs. As expected, a large k_1 implies fast consumption of aryl radical in the grafting process, leading to a low concentration of aryl radical along the length of the main microfluidic chamber (e.g., blue curve in **Supplementary Fig. 10a**). Interestingly, when $k_1 >> 10^0$ (for $k_2 = 10^5$), the grafting is fast enough to completely consume the aryl radical near the HOPG and, therefore, the concentration of radical becomes limited by the rate of diffusion inside the microfluidic device (which does not depend on the value of k_1). This is the case of the simulation with $k_1 = 10^9$ (for $k_2 = 10^5$, red curve in **Supplementary Figs. 10a-b**), whose concentration of aryl radical along the length of the main microfluidic chamber is similar (and completely superimposed) to that obtained with $k_1 = 10^0$ (for $k_2 = 10^5$, blue curve in **Supplementary Figs. 10a-b)**. On the other hand, when $k_1 << 10^0$ (e.g. 10^{-6}, purple curve in **Supplementary Fig. 10a**), the consumption of aryl radical in the grafting is slow, thus resulting in concentrations of aryl radical that increase along the length of the microfluidic channel (purple curve in **Supplementary Figs. 10a-b**), despite some deactivation by I_2. Moreover, values of k_1 of 10^{-3} or larger (e.g. green or blue curves in **Supplementary Figs. 10a-b**), originate concentrations of aryl radical that increase until zone 2, and then decrease until zone 3, as the rate of deactivation starts surpassing the rate of radical formation.

The value of k_2 dictates rate of formation of I_2, i.e. the species responsible for the deactivation of aryl radical. As expected, a high k_2 implies fast deactivation of the aryl radical, and thus a low concentration along the length of the microfluidic chamber (e.g. red curve in **Supplementary Figs. 11a-b**). More importantly, the value of k_2 strongly affects the variation of the radical concentration along the different zones of the microfluidic channel. On one hand, higher values of k_2 imply zones 2 and 3 with lower radical concentrations than zone 1, because the fast formation of I_2 near the inlet of the main microfluidic chamber induces significant deactivation of aryl radical along the chamber (red curve, **Supplementary Fig. 11b**). On the other hand, lower values of k_2 result in a higher concentration in zone 3 than in zones 1 and 2 because the slow formation of I_2 induces little deactivation of aryl radical in the main microfluidic chamber (green curve, **Supplementary Fig. 11b**). Between these scenarios, there are values of k_2 that lead to a higher concentration in zone 2 than in zones 1 and 3, as the formation of I_2 occurs throughout the channel (blue curve, **Supplementary Fig. 11b**).

The k_1 and k_2 values fitting the experimental data of layer thickness should lead to concentration of aryl radical increasing from zone 1 to zone 2, and decreasing from zone 2 to zone 3, as that would be consistent with layer thicknesses observed in the experiments (Figure 3b). From the 28 combinations tested (**Supplementary Table 1)**, there are multiple combinations that fit this criterion (**Supplementary Figs. 10-13**). However, most of these combinations only do so marginally, in the sense that they lead to very small increase/decrease between the different zones, that likely would not explain the significant differences in the observed layer thicknesses (see blue and green curves in **Supplementary Fig. 12** showing a slight decrease in radical concentration from zone 2 to zone 3). Furthermore, the concentration profiles were found to be quite sensitive to the value of k_2, with a change of less than one order of magnitude in k_2 resulting in clearly different concentration profiles along the length of the main microfluidic channel (red and blue curves in **Supplementary Fig. 13**). Our numerical results suggest that the reaction rate constants that best fit the experimental data are $k_1 = 10^{-3}$ m·s^{-1} and $k_2 = 2 \times 10^5$ M^{-1}·s^{-1} (combination 15 in **Supplementary Table 1**), as they result in a clear increase in the aryl radical concentration from zone 1 to zone 2, and a similar decrease from zone 2 to zone 3 (blue curve in **Supplementary Fig. 13**). Moreover, they generate curves of aryl radical concentration along the main reactor (both along its length (y axis, **Fig. 2d**) and its width (x axis; **Fig. 2f**)) that are most consistent with the experimentally-obtained thickness of the grafted layer (**Fig. 3b**). Furthermore, this combination of k_1 and k_2 was also found to fit the experimental data for other flow rates (**Supplementary Fig. 23**), which further supports its use to model the dynamics of the HOPG grafting reactions. For this reason, $k_1 = 10^{-3}$ m·s^{-1} and $k_2 = 2 \times 10^5$ M^{-1}·s^{-1} were used in the numerical simulations reported in the main text of the manuscript. In any case, and despite the obtained consistency between the predicted aryl radical concentrations and the experimentally-observed layer thicknesses, it is important to note that there might be other (k_1, k_2) pairs that also fit the experimentally-observed layer thicknesses.

Supplementary References

1. White, F. M. *Viscous fluid flow*. (McGraw-Hill, 1974).
2. Versteeg, H. K. & Malalasekera, W. *An introduction to computational fluid dynamics: the finite volume method*. (Pearson Education Ltd, 2007).
3. Crank, J. *The mathematics of diffusion*. (Oxford university press, 1957).
4. Luong, T. K. N. *et al.* Multinuclear Diffusion NMR Spectroscopy and DFT Modeling: A Powerful Combination for Unraveling the Mechanism of Phosphoester Bond Hydrolysis Catalyzed by Metal-Substituted Polyoxometalates. *Chem. Eur. J.* **21**, 4428–4439 (2015).
5. Galli, C. Radical reactions of arenediazonium ions: An easy entry into the chemistry of the aryl radical. *Chem. Rev.* **88**, 765–792 (1988).
6. Kryger, R. G., Lorand, J. P., Stevens, N. R. & Herron, N. R. Radicals and scavengers. 7. Diffusion controlled scavenging of phenyl radicals and absolute rate constants of several phenyl radical reactions. *J. Am. Chem. Soc.* **99**, 7589–7600 (1977).
7. Kamholz, A. E., Weigl, B. H., Finlayson, B. A. & Yager, P. Quantitative Analysis of Molecular Interaction in a Microfluidic Channel: The T-Sensor. *Anal. Chem.* **71**, 5340–5347 (1999).
